# Heterocyclic Aromatic Amines and Risk of Kidney Stones: A Cross-Sectional Study in US Adults

**DOI:** 10.3389/fpubh.2022.935739

**Published:** 2022-07-14

**Authors:** Guangyuan Zhang, Xiangyu Zou, Weipu Mao, Ming Chen

**Affiliations:** ^1^Department of Urology, Affiliated Zhongda Hospital of Southeast University, Nanjing, China; ^2^School of Basic Medical Sciences, Weifang Medical University, Weifang, China

**Keywords:** kidney stone, heterocyclic aromatic amines, Harman, NHANES, propensity score matching

## Abstract

**Background:**

Heterocyclic aromatic amines (HAAs) are a group of harmful substances produced while cooking meat at high temperatures. This study aimed to investigate the relationship between HAAs and the occurrence of kidney stones.

**Methods:**

Data on the level of four HAAs, including 2-Amino-9H-pyrido [2, 3-b] indole (A-α-C), 1-Methyl-9H-pyrido [3, 4-b] indole (Harman), 9H-Pyrido [3, 4-b] indole (Norharman), and 2-Amino-1-methyl-6-phenylimidazo [4, 5-b] pyridine (PhIP), in the urine from adult participants were extracted from the 2013–2014 NHANES database. Propensity score matching (PSM) was used to balance confounding variables between the stone former and non-stone former groups, and logistic regression analysis was performed to analyze the relationship between HAAs and the occurrence of kidney stones.

**Results:**

Of the 1,558 eligible participants, a history of kidney stones was self-reported by 140 (9.0%). Compared to non-stone formers, stone formers had higher concentrations of A-α-C, Harman, and Norharman and lower concentrations of PhlP in urine. After adjusting for all other confounding variables, multivariate logistic regression analysis showed that the high-Harman group had a higher risk of kidney stones than the low-Harman group [adjusted odds ratios (aOR) = 1.618, 95% CI: 1.076–2.433, *p* = 0.021]. After PSM analysis, Harman concentration remained a risk factor for kidney stones (high-Harman group vs. low-Harman group: aOR = 1.951, 95% CI: 1.059–3.596, *p* = 0.032).

**Conclusion:**

Increased urinary Harman concentrations are associated with an increased risk of kidney stones in the general US population.

## Introduction

Kidney stones are a common type of urological disease, accounting for 40–50% of all urinary stone diseases (1). The presence of kidney stones varies depending on geography, race, and lifestyle habits. The presence of kidney stones in developed countries is 5–15%, and the presence of kidney stones in China is gradually increasing, with a prevalence of 6.4% in adults and a younger age of onset (2, 3). Kidney stones are mineral deposits in the junction of renal calyces, renal pelvis, or pelvic ureter that cause hematuria and back pain, leading to urological infection, urinary tract obstruction, and even uremia (4, 5).

The pathogenesis of kidney stones is more complicated, with more predisposing variables such as dietary, metabolic, pharmacological, and environmental factors. Additionally, genetic mutations play an important role in the formation of kidney stones (6, 7). Meat intake is prevalent in the Western diet, and high-temperature cooking of meat products produces mutagenic and carcinogenic chemicals such as lipid and protein oxidation products, heterocyclic aromatic amines (HAAs), and polycyclic aromatic hydrocarbons (PAHs) (8–11). In the 1970s, HAAs, a class of mutagenic compounds, were detected in cooked meat (12). HAAs are a class of nitrogen-containing heterocyclic hazardous substances, and studies have found that more than 25 HAAs are formed in cooked red meat (13, 14). In the Maillard reaction system, amino acids, sugars, and creatine are the main components involved in the formation of such culinary toxicants (15). Our previous study found that increased concentrations of PAHs in urine were associated with an increased risk of kidney stones (16). However, no studies have examined the relationship between HAAs and the occurrence of kidney stones.

In the current study, we used data from the 2013–2014 National Health and Nutrition Examination Survey (NHANES) database to explore the relationship between urinary concentrations of HAAs and the risk of kidney stones, which was validated by propensity score matching (PSM) analysis.

## Patients and Methods

### Data Sources and Preparation

Data for this study were obtained from the NHANES database, which is a cross-sectional, nationally based survey conducted in the US to assess the health and nutritional status of adults and children. The interview component of the NHANES database included demographic information, socioeconomic information, dietary status, and health-related questions. The examination component included medical health-related physical examinations and laboratory tests. Beginning in 1999, the NHANES database publishes data files from the survey online on a 2-year cycle (17). All NHANES protocols were reviewed and approved by the National Center for Health Statistics (NCHS) Ethics Committee, and written informed consent was obtained from relevant participants for any data collection.

The 2013–2014 cycle contains data on HAAs. A total of 5,769 adults (≥20 years) participated in the survey during 2013–2014. The following exclusion criteria were included: (a) incomplete stone survey (*n* = 11); (b) unknown/abnormal HAAs (*n* = 4,023); (c) unknown body mass index (BMI), family income, education level, diabetes, renal function (creatinine, blood urea nitrogen, and uric acid levels) and urinary creatinine (*n* = 168). Based on these criteria, a total of 1,558 study subjects were finally included in this study.

### Study Variables and Definitions

The presence of kidney stones was determined based on the KIQ026 question from the Kidney Conditions-Urology survey in the questionnaire data, which was “Have you ever had a kidney stone?” and participants who answered “yes” were defined as having a history of kidney stones.

Concentrations of HAAs in urine were measured by isotope-dilution high-performance liquid chromatography/electrospray ionization tandem mass spectrometry (ID HPLC-ESI MS/MS). The urine samples were fortified and hydrolyzed at 70°C under alkaline conditions for 5 h. The samples were then extracted by solid-phase extraction, after which the analytes were eluted and analyzed by LC/MS/MS (https://wwwn.cdc.gov/Nchs/Nhanes/2013-2014/HCAAS_H.htm). Only the following 10 HAAs appeared in the 2013–2014 cycle: 2-Amino-9H-pyrido [2, 3-b] indole (A-α-C), 2-Amino-3-methyl-9H-pyriodo [2, 3-b] indole (MeA-α-C), 1-Methyl-9H-pyrido [3, 4-b] indole (Harman), 9H-Pyrido [3, 4-b] indole (Norharman), 2-Amino-6-methyldipyrido [1, 2-a:3', 2'-d] imidazole (Glu-P-1), 2-Aminodipyrido [1, 2-a:3', 2'-d] imidazole (Glu-P-2), 3-Amino-1,4-dimethyl-5H-pyrido [4, 3-b] indole (Trp-P-1), 1-Methyl-3-amino-5H-pyrido [4, 3-b] indole (Trp-P-2), 2-Amino-3-methyl-3H-imidazo [4, 5-f] quinoline (IQ), and 2-Amino-1-methyl-6-phenylimidazo [4, 5-b] pyridine (PhIP). The lower limit of detection (LOD) for the 10 HAAs was 0.62, 0.33, 4.59, 12.6, 0.31, 0.83, 0.79, 0.63, 0.37, and 0.34 pg/mL, respectively. Since the percentage of ≥ LOD for Glu-P-1, Glu-P-2, IQ, MeA-α-C, Trp-P-1, and Trp-P-2 was below 20%, further analysis was not considered. In addition, age, sex, marital status, race, family income, education level, BMI, hypertension, diabetes, physical activities, smoking status, urinary creatinine, and renal function [creatinine, blood urea nitrogen, uric acid levels, and estimated glomerular filtration rate (eGFR)] were also included in the study. The BMI was calculated as weight (kg)/[height (m2)^*^height (m2)]. Hypertension and diabetes mellitus was diagnosed by a physician or other health professional. The estimated glomerular filtration rate (eGFR) was measured in our previous study (5, 18).

### Statistical Analysis

The categorical data were described by frequency (*n*) and percentage (%), and continuous data were described by the mean ± *SD*. In processing the data, a weighted analysis the data was performed. HAAs were divided into high and low concentration groups according to the median. A univariate and multivariate logistic regression was performed to evaluate the association between different concentration groups of HAAs and kidney stones, and the results were expressed as adjusted odds ratios (aORs) and 95% CI intervals, and the same analysis was performed in all subgroups. We constructed three multivariate logistic regression models: the base model included participant demographic variables, including age, sex, race, education level, marital status, and family income; the core model added variables related to participants' living conditions, such as BMI, diabetes, hypertension, physical activity status, and smoking status; and the extended model added creatinine, blood urea nitrogen, uric acid, eGFR, and urinary creatinine, which are indicators related to renal function.

Additionally, a 1:1 PSM analysis was performed to balance the differences between stone former and non-stone former groups, with the following adjusted confounding variables: age, sex, race, education level, marital status, family income, BMI, hypertension, diabetes, physical activity status, and smoking status. To further test the correctness of the results, the data after PSM were reanalyzed. All data and figures in this study were organized and analyzed using R software (version 3.5.3) and SPSS software (version 24). Statistical significance was set at *p* < 0.05.

## Results

**Figure 1:**
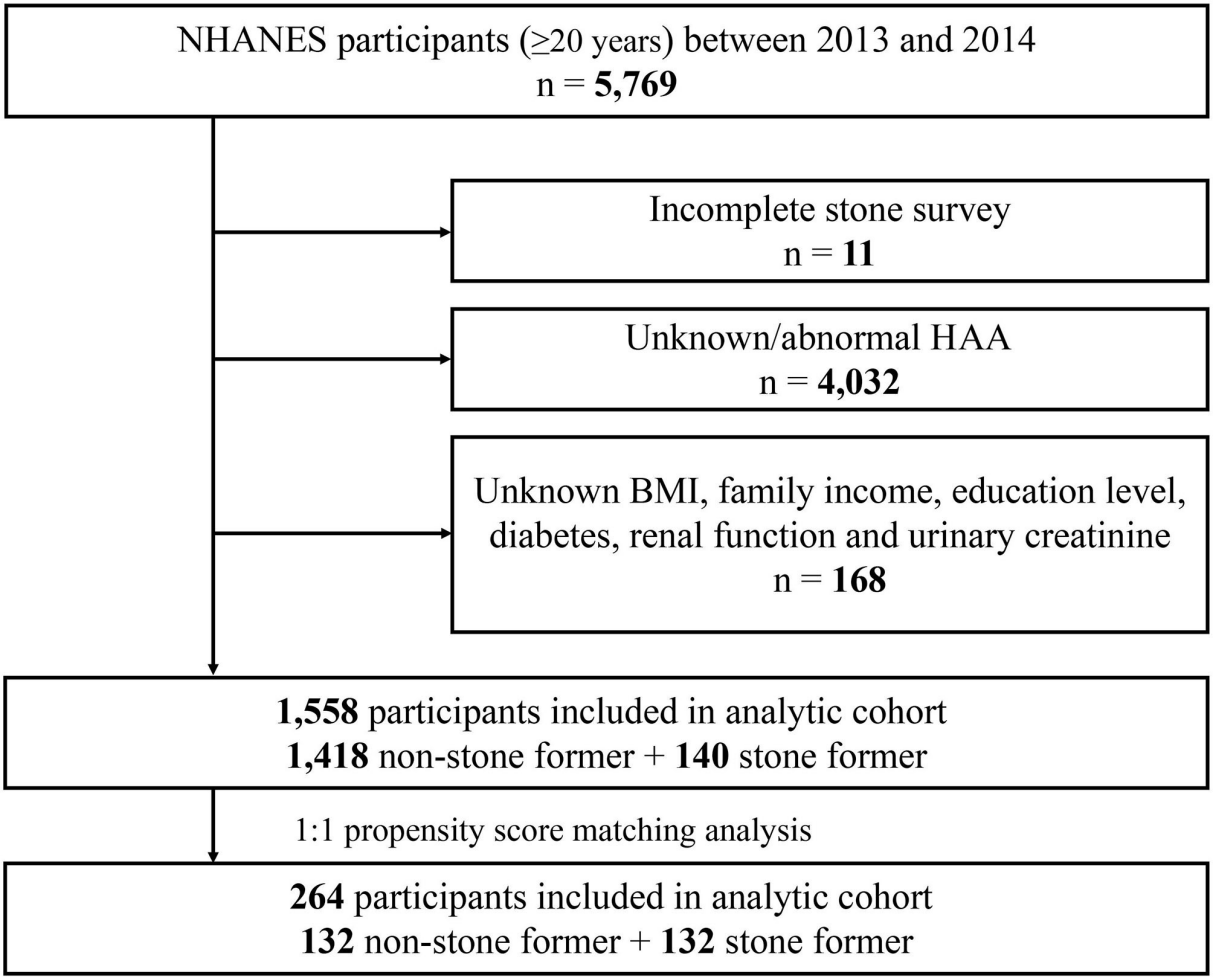
Schematic flow diagram of inclusion and exclusion criteria.

Based on the criteria in the flow chart (Figure 1), 1,558 participants were eventually included in the analysis, of whom 140 (9%) self-reported history of kidney stones, and 1,418 (91%) had no history of kidney stones. The clinicopathological characteristics of all participants are shown in Table 1. A chi-square test revealed significant differences between the stone former and the non-stone former groups in six variables: age, race, BMI, hypertension, diabetes, and physical activity (all *p* < 0.05). In addition, the stone former group had higher levels of blood urea nitrogen, blood creatinine, uric acid, urine creatinine, and lower levels of eGFR compared with the non-stone former group.

**Table 1:** Baseline characteristics of NHANES participants between 2013 and 2014 before PSM.

**Characteristic**	**Total**	**None-stone formers**	**Stone formers**	***P***
	**No. (%)**	**No. (%)**	**No. (%)**	
*N*	1,558	1,418 (91.0)	140 (9.0)	
Gender				0.334
Male	774 (49.7)	699 (49.3)	75 (53.6)	
Female	784 (50.3)	719 (50.7)	65 (46.4)	
Age, years				<0.001
<50	843 (54.1)	795 (56.1)	48 (34.3)	
≥50	715 (45.9)	623 (43.9)	92 (65.7)	
Race				<0.001
Non-Hispanic white	689 (44.2)	598 (42.2)	91 (65.0)	
Non-Hispanic black	301 (19.3)	285 (20.1)	16 (11.4)	
Mexican American	217 (13.9)	203 (14.3)	14 (10.0)	
Other Hispanic	135 (8.7)	124 (8.7)	11 (7.9)	
Other	216 (13.9)	208 (14.7)	8 (5.7)	
Marital status				0.170
Married	793 (50.9)	714 (50.4)	79 (56.4)	
Unmarried	765 (49.1)	704 (49.6)	61 (43.6)	
Education				0.509
Less than high school	315 (20.2)	282 (19.9)	33 (23.6)	
High school or equivalent	344 (22.1)	312 (22.0)	32 (22.9)	
College or above	899 (57.7)	824 (58.1)	75 (53.6)	
Family income				0.118
$0–$19,999	335 (21.5)	299 (21.1)	36 (25.7)	
$20,000–$44,999	457 (29.3)	413 (29.1)	44 (31.4)	
$45,000–$74,999	296 (19.0)	266 (18.8)	30 (21.4)	
≥$75,000	470 (30.2)	440 (31.0)	30 (21.4)	
BMI (kg/m^2^)				0.002
<25.0	477 (30.6)	450 (31.7)	27 (19.3)	
≥25.0	1,081 (69.4)	968 (68.3)	113 (80.7)	
Hypertension				<0.001
Yes	552 (35.4)	478 (33.7)	74 (52.9)	
No/unknown	1,006 (64.6)	940 (66.3)	66 (47.1)	
Diabetes				<0.001
Yes	174 (11.2)	145 (10.2)	29 (20.7)	
No	1,333 (85.6)	1,229 (86.7)	104 (74.3)	
Borderline	51 (3.3)	44 (3.1)	7 (5.0)	
Physical activities				<0.001
No	774 (49.7)	701 (49.4)	73 (52.1)	
Vigorous	134 (8.6)	128 (9.0)	6 (4.3)	
Moderate	650 (41.7)	589 (41.5)	61 (43.6)	
Smoking				0.390
Never	890 (57.1)	817 (57.6)	73 (52.1)	
Former	358 (23.0)	320 (22.6)	38 (27.1)	
Current	310 (19.9)	281 (19.8)	29 (20.7)	
Blood urea nitrogen (mg/dL)	13.11 ± 5.56	13.00 ± 5.57	14.19 ± 5.41	0.016
Creatinine (mg/dL)	1.01 ± 0.25	1.01 ± 0.24	1.03 ± 0.32	0.355
Uric acid (mg/dL)	5.43 ± 1.40	5.42 ± 1.41	5.63 ± 1.30	0.086
eGFR [mL/(min·1.73 m^2^)]	83.41 ± 24.74	84.29 ± 24.70	74.44 ± 23.46	<0.001
Urinary creatinine (mg/dL)	109.9 ± 72.0	109.7 ± 72.6	111.7 ± 66.5	0.752

*a For categorical variables, P-values were analyzed by chi-square tests. For continuous variables, data were described by the mean ± SD and the t-test for the slope was used in generalized linear models. NHANES, National Health and Nutrition Examination Survey; BMI, body mass index; eGFR, estimated glomerular filtration rate; PSM, propensity score matching*.

Table 2 demonstrates the concentrations of HAAs in the urine of stone formers and non-stone formers. Since the percentage of ≥ LOD for Glu-P-1, Glu-P-2, IQ, MeA-α-C, Trp-P-1, and Trp-P-2 was below 20%, further analysis was not considered. We found higher concentrations of A-α-C, Harman, and Norharman and lower concentrations of PhlP in the urine of stone formers than in non-stone formers.

**Table 2:** The distribution levels of urinary HAAs in the study population with and without kidney stones in NHANES 2013–2014.

**HAAs**	**≥LOD**	**Total study population**	**None-stone formers**	**Stone formers**
	**(%)**	**Mean ±SD**	**Mean ±SD**	**Mean ±SD**
A-a-C	53.1	11.00 ± 28.10	10.57 ± 27.80	15.30 ± 30.78
Glu-P-1	2.0	0.23 ± 0.11	0.23 ± 0.10	0.26 ± 0.22
Glu-P-2	0.3	0.59 ± 0.05	0.59 ± 0.03	0.60 ± 0.13
Harman	99.9	244.2 ± 419.2	242.63 ± 423.03	260.3 ± 379.2
IQ	1.9	0.28 ± 0.27	0.28 ± 0.28	0.28 ± 0.12
MeA-a-C	20.0	0.81 ± 1.87	0.79 ± 1.86	0.93 ± 2.00
Norharman	99.9	493.4 ± 519.8	488.4 ± 513.7	543.4 ± 578.0
PhlP	57.5	3.76 ± 11.98	3.84 ± 12.33	2.98 ± 7.50
Trp-P-1	1.9	0.60 ± 0.35	0.59 ± 0.31	0.63 ± 0.63
Trp-P-1	2.1	0.47 ± 0.24	0.47 ± 0.25	0.47 ± 0.12

*Concentration, pg/mL. HAAs, heterocyclic aromatic amines; NHANES, National Health and Nutrition Examination Survey; LOD, limits of detection; SD, standard deviation*.

**Figure 2:**
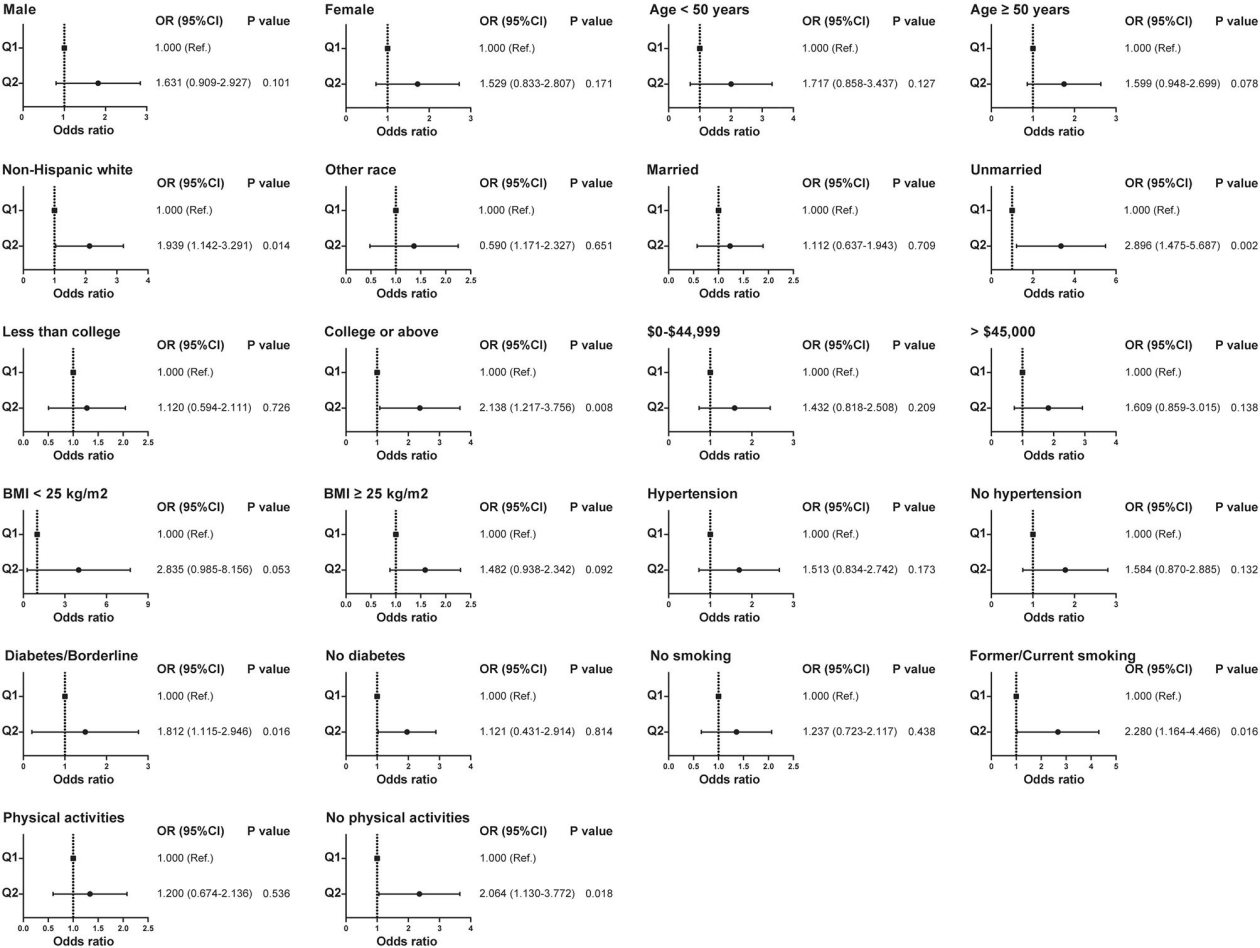
Relationship between Harman and kidney stones in subgroups with different clinical characteristics before propensity score matching.

Patients were categorized into high and low concentration groups according to the median level of HAAs. Logistic regression was performed to analyze the relationship between HAAs and the occurrence of kidney stones. Univariate logistic regression analysis showed that the risk of kidney stones was 65.2% (95% CI: 1.157–2.358, *p* = 0.006) higher in the high Harman group compared to that in the low Harman group; 66.6% (95% CI: 1.167–2.378, *p* = 0.005) higher in the high Norharman group compared to the low Norharman group. Multivariate logistic regression analysis that high Harman was an independent risk factor for kidney stones in both the base model (aOR = 1.511, 95% CI: 1.041–2.194, *p* = 0.03), core model (aOR = 1.622, 95% CI: 1.106–2.38, *p* = 0.013), and expanded model (aOR = 1.618, 95% CI: 1.076–2.433, *p* = 0.021) (Table 3). In addition, all subgroup analyses also found an increased risk of kidney stones in the high Harman group compared to the low Harman group (Figure 2).

**Table 3:** Adjusted odds ratios for associations between the urinary HAAs and the presence of kidney stones in NHANES 2013–2014 before PSM.

**HAAs**	**Univariate analysis**		**Basic model**		**Core model**		**Extended model**	
	**aOR (95% CI)**	***P***	**aOR (95% CI)**	***P***	**aOR (95% CI)**	***P***	**aOR (95% CI)**	***P***
A-a-C								
Low group	1.000		1.000		1.000		1.000	
High group	1.059 (0.748–1.499)	0.747	1.116 (0.771–1.616)	0.562	1.196 (0.789–1.812)	0.398	1.172 (0.765–1.795)	0.467
Harman								
Low group	1.000		1.000		1.000		1.000	
High group	1.652 (1.157–2.358)	0.006	1.511 (1.041–2.194)	0.030	1.622 (1.106–2.380)	0.013	1.618 (1.076–2.433)	0.021
Norharman								
Low group	1.000		1.000		1.000		1.000	
High group	1.666 (1.167–2.378)	0.005	1.434 (0.990–2.076)	0.057	1.441 (0.987–2.104)	0.058	1.397 (0.934–2.088)	0.103
PhlP								
Low group	1.000		1.000		1.000		1.000	
High group	1.118 (0.790–1.583)	0.529	1.290 (0.897–1.853)	0.170	1.330 (0.916–1.931)	0.133	1.355 (0.916–2.004)	0.129

*Adjusted covariates: Basic model: gender, age, race, marital status, education levels, and family income; Core model: basic model plus BMI, hypertension, diabetes, physical activities, and smoking; Extended model: core model plus blood urea nitrogen, creatinine, uric acid, eGFR, and urinary creatinine. NHANES, National Health and Nutrition Examination Survey; PSM, propensity score matching; HAAs, heterocyclic aromatic amines; BMI, body mass index; eGFR, estimated glomerular filtration rate; CI: confidence interval; aOR: adjusted odds ratio*.

**Figure 3:**
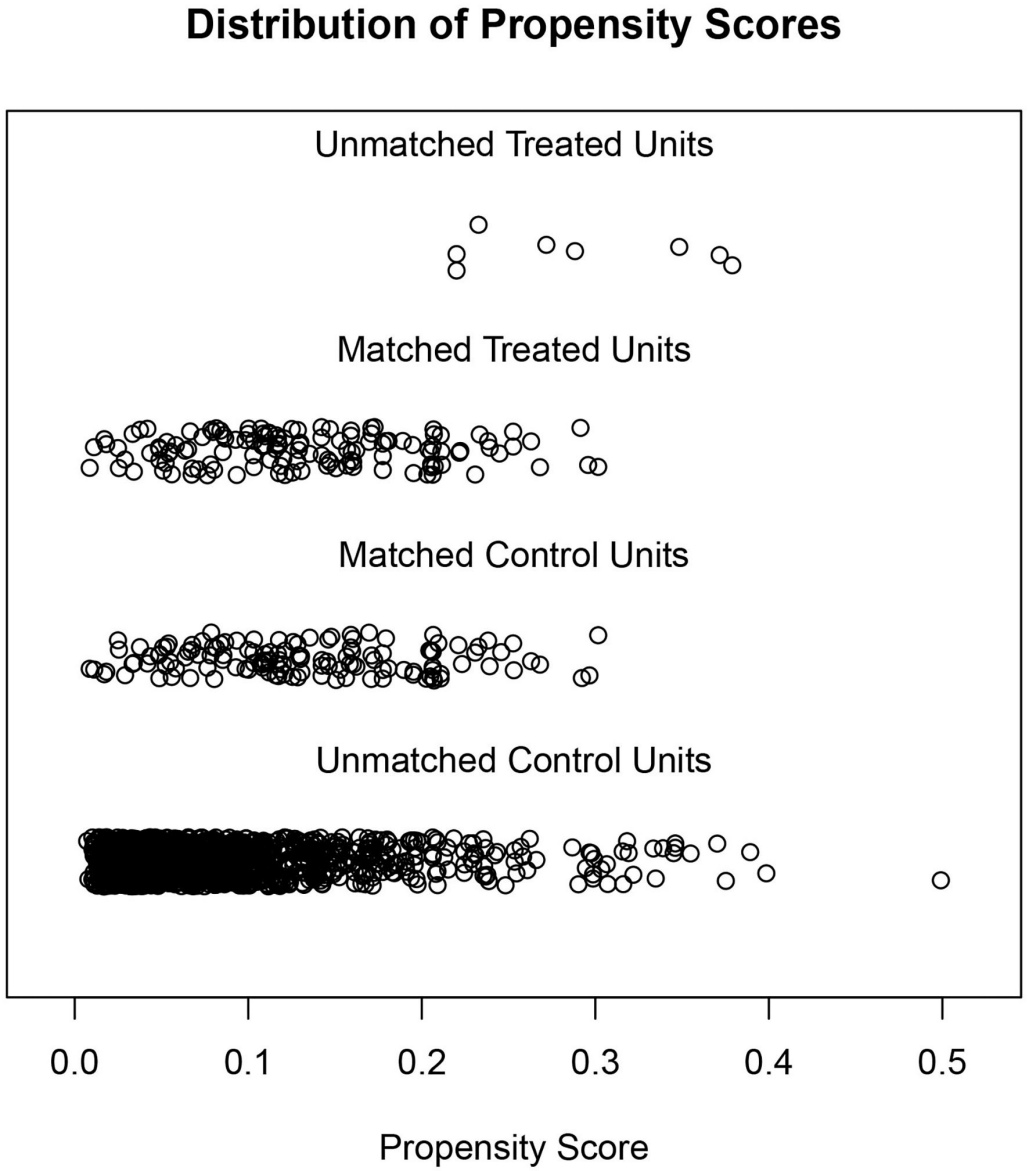
Distribution of the stone former and non-stone former groups before and after propensity score matching.

**Figure 4:**
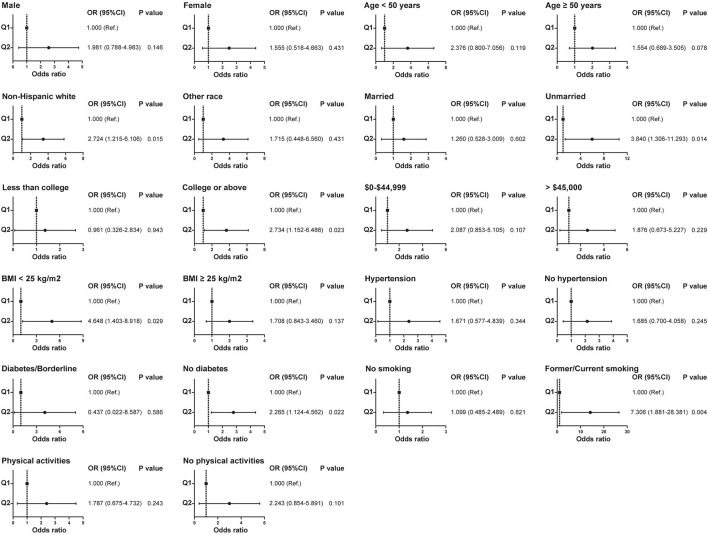
Relationship between Harman and kidney stones in subgroups with different clinical characteristics after propensity score matching.

Due to the differences in some variables between the stone former and non-stone former groups, we used a 1:1 PSM to correct for differences between the two groups (Figure 3 and Supplementary Figure S1). A total of 132 participants were included in the stone former and non-stone former groups, respectively. The clinicopathological characteristics of all participants after PSM are shown in Table 4. We found higher concentrations of A-α-C, Harman, and Norharman and lower concentrations of PhlP in the urine of participants in the stone former group than in the non-stone former group; however, the differences were not statistically significant. We likewise performed a multivariate logistic regression analysis and found that high Harman was an independent risk factor (aOR = 1.951, 95% CI: 1.059–3.596, *p* = 0.032) for kidney stones (Table 5), and similar results were found for subgroup analysis (Figure 4).

**Table 4:** Baseline characteristics of NHANES participants between 2013 and 2014 after PSM.

**Characteristic**	**Total**	**None-stone formers**	**Stone formers**	***P***
	**No. (%)**	**No. (%)**	**No. (%)**	
N	264	132 (50.0)	132 (50.0)	
Gender				0.216
Male	146 (55.3)	78 (59.1)	68 (51.5)	
Female	118 (44.7)	54 (40.9)	64 (48.5)	
Age, years				0.898
<50	97 (36.7)	49 (37.1)	48 (36.4)	
≥50	167 (63.3)	83 (62.9)	84 (63.6)	
Race				0.758
Non-Hispanic white	174 (65.9)	91 (68.9)	83 (62.9)	
Non-Hispanic black	29 (11.0)	13 (9.80	16 (12.1)	
Mexican American	29 (11.0)	15 (11.4)	14 (10.6)	
Other Hispanic	19 (7.2)	8 (6.1)	11 (8.3)	
Other	13 (4.9)	5 (3.8)	8 (6.1)	
Marital status				0.902
Married	143 (54.2)	72 (54.5)	71 (53.8)	
Unmarried	121 (45.8)	60 (45.5)	61 (46.2)	
Education				0.827
Less than high school	60 (22.7)	28 (21.2)	32 (24.2)	
High school or equivalent	62 (23.5)	31 (23.5)	31 (23.5)	
College or above	142 (53.8)	73 (55.3)	69 (52.3)	
Family income				0.554
$0–$19,999	75 (28.4)	41 (31.1)	34 (25.8)	
$20,000–$44,999	80 (30.3)	38 (28.8)	42 (31.8)	
$45,000–$74,999	51 (19.3)	22 (16.7)	29 (22.0)	
≥$75,000	58 (22.0)	31 (23.5)	27 (20.5)	
BMI (kg/m^2^)				0.878
<25.0	53 (20.1)	27 (20.5)	26 (19.7)	
≥25.0	211 (79.9)	105 (79.5)	106 (80.3)	
Hypertension				0.622
Yes	128 (48.5)	62 (47.0)	66 (50.0)	
No/unknown	136 (51.5)	70 (53.0)	66 (50.0)	
Diabetes				0.534
Yes	38 (14.4)	16 (12.1)	22 (16.7)	
No	213 (80.7)	110 (83.3)	103 (78.0)	
Borderline	13 (4.9)	6 (4.5)	7 (5.3)	
Physical activities				0.472
No	135 (51.1)	69 (52.3)	66 (50.0)	
Vigorous	16 (6.1)	10 (7.6)	6 (4.5)	
Moderate	113 (42.8)	53 (40.2)	60 (45.5)	
Smoking				0.885
Never	140 (53.0)	72 (54.5)	68 (51.5)	
Former	68 (25.8)	33 (25.0)	35 (26.5)	
Current	29 (21.2)	27 (20.5)	29 (22.0)	
Blood urea nitrogen (mg/dL)	14.13 ± 5.23	14.36 ± 5.33	13.90 ± 5.14	0.481
Creatinine (mg/dL)	1.00 ± 0.20	0.99 ± 0.20	1.01 ± 0.20	0.529
Uric acid (mg/dL)	5.69 ± 1.37	5.80 ± 1.46	5.58 ± 1.28	0.210
eGFR [mL/(min·1.73 m^2^)]	75.70 ± 22.15	75.81 ± 21.39	75.58 ± 22.96	0.934
Urinary creatinine (mg/dL)	109.83 ± 66.84	108.22 ± 66.63	111.45 ± 67.27	0.696
A-a-C (pg/mL)	12.83 ± 29.47	9.80 ± 26.96	15.86 ± 31.60	0.095
Harman (pg/mL)	257.7 ± 370.8	247.9 ± 353.3	267.5 ± 388.5	0.668
Norharman (pg/mL)	547.7 ± 605.5	545.3 ± 620.8	550.1 ± 592.0	0.949
PhlP (pg/mL)	3.79 ± 13.49	4.63 ± 17.47	2.85 ± 7.63	0.259

*For categorical variables, P-values were analyzed by chi-square tests. For continuous variables, data were described by the mean ± SD and the t-test for the slope was used in generalized linear models. NHANES, National Health and Nutrition Examination Survey; BMI, body mass index; eGFR, estimated glomerular filtration rate; PSM, propensity score matching*.

**Table 5:** Adjusted odds ratios for associations between the urinary HAAs and the presence of kidney stones in NHANES 2013–2014 after PSM.

**HAAs**	**Univariate analysis**	**Basic model**	**Core model**	**Extended model**
	**aOR (95% CI)**	***P***	**aOR (95% CI)**	***P***	**aOR (95% CI)**	***P***	**aOR (95% CI)**	***P***
A-a-C								
Low group	1.000		1.000		1.000		1.000	
High group	1.237 (0.763–2.006)	0.389	1.301 (0.774–2.184)	0.320	1.248 (0.677–2.298)	0.478	1.255 (0.660–2.387)	0.488
Harman								
Low group	1.000		1.000		1.000		1.000	
High group	1.647 (1.007–2.694)	0.047	1.796 (1.053–3.062)	0.032	1.871 (1.076–3.255)	0.026	1.951 (1.059–3.596)	0.032
Norharman								
Low group	1.000		1.000		1.000		1.000	
High group	1.538 (0.944–2.507)	0.084	1.580 (0.937–2.663)	0.086	1.571 (0.909–2.714)	0.105	1.564 (0.873–2.801)	0.133
PhlP								
Low group	1.000		1.000		1.000		1.000	
High group	1.441 (0.887–2.341)	0.140	1.336 (0.800–2.232)	0.268	1.432 (0.833–2.461)	0.194	1.549 (0.874–2.745)	0.134

*Adjusted covariates: Basic model: gender, age, race, marital status, education levels, and family income; Core model: basic model plus BMI, hypertension, diabetes, physical activities, and smoking; Extended model: core model plus blood urea nitrogen, creatinine, uric acid, eGFR, and urinary creatinine; NHANES, National Health and Nutrition Examination Survey; PSM, propensity score matching; HAAs, heterocyclic aromatic amines; BMI, body mass index; eGFR, estimated glomerular filtration rate; CI, confidence interval; aOR, adjusted odds ratio*.

## Discussion

Kidney stone is a common urological disease that seriously affects human health and imposes a substantial medical burden on the patient's family. Therefore, early identification of risk factors for kidney stones has important clinical significance. In the present study, we used the NHANES database, which is representative of the US population, and found that the concentration of some HAAs (A-α-C, Harman, and Norharman) in the urine of patients with kidney stones was higher than that of patients without kidney stones. In addition, after adjusting for other confounding variables, multivariate logistic regression analysis showed that the concentration of Harman in urine was positively associated with the risk of kidney stones, with a 61.8% increased risk in the high Harman group compared to the low Harman group. To exclude the effects of other confounding variables and to test the validity of these results, we used PSM analysis to adjust for differences between the stone former and non-stone former groups. Subsequent multivariate logistic regression and subgroup analysis confirmed that increased concentrations of Harman in the urine might be a risk factor for kidney stones.

Heterocyclic aromatic amines are a class of polycyclic aromatic compounds that are formed naturally during the heating of protein-based foods such as meat (19). The average exposure to meat HAAs varies by the nation due to differences in the diets of the East and West, especially the amount of meat consumed (20). Studies have shown that the average daily exposure to HAAs is 565.3 ng/d in Americans, 69.4 ng/d in Europeans, and 49.94 ng/d in the Chinese population (21–23). Exposure to meat HAAs is correlated with the presence of kidney stones.

Numerous experiments have shown that HAAs can cause gene mutations or cancer by disrupting hydrogen bonds in DNA strands through the insertion/deletion of gene loci (24, 25). Excessive consumption of foods containing HAAs, especially cooked protein meats, is associated with an increased risk of several cancers (26, 27). Components of HAAs in grilled meat can be transformed into possibly carcinogenic genotoxic chemicals, and human bodies can detoxify HAAs by conjugation and eliminate the metabolites through urine (28, 29). Urinary HAAs are biomarkers that can be used to determine the exposure and the relative biological activity of detoxification in the organism (30).

Oxidative stress is closely associated with the formation of kidney stones (31). Lawana et al. (32) found that low doses of Harmane caused a 1.6-fold increase in reactive oxygen species (ROC) production in SH-SY5Y cells, which increased to 3.5-fold at higher doses. Furthermore, there was a dose-dependent increase in ROC synthesis in SH-SY5Y cells in response to Phlp and its metabolites. In addition, increased exposure to HAAs was associated with increased dopamine oxidation metabolites *in vivo*, which in turn caused oxidative stress in cells (33). Our findings provide a potential clinical basis for investigating the relationship between HAAs and the presence of kidney stones, and the accumulation of HAAs may increase the presence of kidney stones through increased oxidative stress.

Several findings of the current study are noteworthy. First, the participants included in this study were representative of the national population of the US, and the findings warrant generalization. Second, we constructed multiple multivariate logistic regression analysis models, adjusting for other confounding variables separately in the statistical analysis. Finally, to verify the reliability of our findings, we also conducted a 1:1 PSM analysis, which was used to eliminate the effects caused by quantitative differences between groups. However, there are certain limitations to the study. First, the NHANES database is a cross-sectional retrospective questionnaire with its inherent limitations. Second, the type of kidney stones is not available in the NHANES database, and different stone types may yield different results. Finally, the causal relationship between the concentration of urinary HAAs and kidney stones is difficult to determine, thus warranting further basic experiments for determination.

## Conclusion

Higher concentrations of urinary HAAs are associated with the development of kidney stones, and increased urinary Harman concentration may be a risk factor for kidney stones.

## Data Availability Statement

The datasets presented in this study can be found in online repositories. The names of the repository/repositories and accession number(s) can be found in the article/Supplementary Material.

## Ethics Statement

The studies involving human participants were reviewed, approved and was carried out in accordance with the recommendations of NHANES committee with written informed consent from all subjects. All subjects gave written informed consent in accordance with the Declaration of Helsinki. The protocol was approved by the NHANES committee. The patients/participants provided their written informed consent to participate in this study.

## Author Contributions

All authors contributed to data analysis, drafting or revising the article, have agreed on the journal to which the article will be submitted, gave final approval of the version to be published, and agree to be accountable for all aspects of the work.

## Funding

This work was supported by National Natural Science Foundation of China (81900618, 82170703), Tai-Shan Scholar Program from Shandong Province (tsqn202103116), Jiangsu Provincial Key Research and Development Program (BE2019751), Innovative Team of Jiangsu Provincial (2017ZXKJQW07), and the National Key Research and Development Program of China (SQ2017YFSF090096).

## Conflict of Interest

The authors declare that the research was conducted in the absence of any commercial or financial relationships that could be construed as a potential conflict of interest.

## Publisher's Note

All claims expressed in this article are solely those of the authors and do not necessarily represent those of their affiliated organizations, or those of the publisher, the editors and the reviewers. Any product that may be evaluated in this article, or claim that may be made by its manufacturer, is not guaranteed or endorsed by the publisher.

## Supplementary Material

Supplementary Figure S1 The proportion of the stone former and non-stone former groups before and after propensity score matching.
