# Supplementary figures and images for: Heterocyclic Aromatic Amines and Risk of Kidney Stones: A Cross-Sectional Study in US Adults
Source: Front Public Health. 2022 Jul 14;10:935739. doi: 10.3389/fpubh.2022.935739 (PMC9330616; doi:10.3389/fpubh.2022.935739)

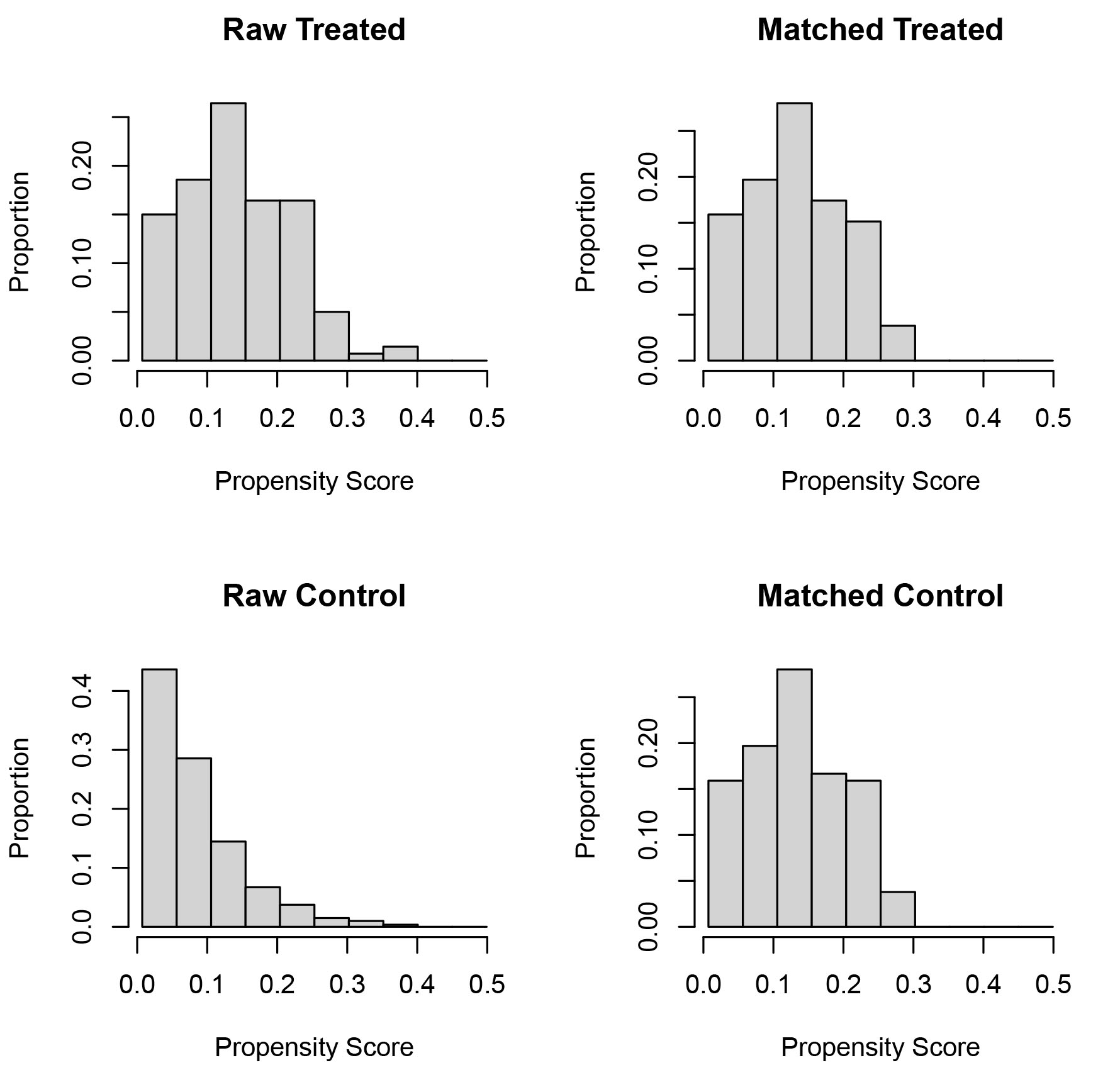

Supplement: Supplementary Figure S1 — The proportion of the stone former and non-stone former groups before and after propensity score matching. [file Image_1.JPEG]
